# CAMP-negative group B *Streptococcus* in pregnant women: molecular and clinical features with implications for diagnostics and neonatal management

**DOI:** 10.1007/s10096-026-05483-8

**Published:** 2026-03-27

**Authors:** Lianfen Huang, Zeying Lin, Sufei Zhu, Chengman Fong, Zengyu Tan, Kankan Gao, Manqiu Kang, Zimeng Liu, Xiaolan Chen, Huamin Zhong, Yongqiang Xie, Bingshao Liang, Fei Gao, Yan Long

**Affiliations:** Department of Laboratory Medicine, Guangdong Provincial Clinical Research Center for Laboratory Medicine, Guangzhou Medical University Affiliated Women and Children’s Medical Center, Guangzhou, China

**Keywords:** Group B *Streptococcus*, CAMP test, Serotype, Virulence genes, Antimicrobial resistance

## Abstract

**Objectives:**

The Christie-Atkins-Munch-Peterson (CAMP) test and *cfb*-targeted polymerase chain reaction (PCR) are widely used for identification of *Streptococcus agalactiae* (Group B *Streptococcus*, GBS). However, CAMP-negative strains may evade detection. This study aimed to characterize antimicrobial resistance, molecular profiles, and pregnancy outcomes associated with CAMP-negative GBS.

**Methods:**

55 CAMP-negative GBS strains (45 from pregnant women and 10 from non-pregnant outpatients) were collected; the 10 non-pregnant isolates were included only for molecular characterization and antimicrobial resistance profiling, while clinical pregnancy outcomes were analyzed exclusively in the pregnant cohort. A total of 66 contemporaneous CAMP-positive controls were collected (2017–2021) at Guangzhou Medical University Affiliated Women and Children’s Medical Center. Isolates were identified by VITEK-2 (bioMérieux) or matrix-assisted laser desorption/ionization time-of-flight mass spectrometry (MALDI-TOF MS) followed by CAMP test. Pregnancy outcomes were retrieved. Antimicrobial susceptibility, multilocus sequence typing (MLST), capsular serotyping, and 11 virulence genes were analyzed.

**Results:**

Among 46,746 pregnant women screened, 6,108 (13.1%) were GBS-positive, includin*g* 45 (0.74%) CAMP-negative isolates. Adverse outcomes were observed in 42.2% (19/45), including premature rupture of membranes (11/45, 24.4%), low birth weight (6/45, 13.3%), and preterm delivery (2/45, 4.4%). All isolates were susceptible to β-lactams, vancomycin, linezolid, and levofloxacin, but resistant to erythromycin (18/55, 32.7%), clindamycin (31/55, 56.4%), and tetracycline (35/55, 63.6%). CAMP-negative isolates were predominantly of serotype III and sequence type (ST) 862 (50/55, 90.9%), compared with diverse types in controls. All lacked *cfb* and *bac* genes but harbored 9 remaining virulence genes.

**Conclusions:**

Although CAMP-negative GBS is rare in clinical settings, it can escape detection by the CAMP test and single-target PCR assays targeting the *cfb* gene, leading to false negative results. Integrated diagnostic strategies combining culture-based screening with multiplex PCR are therefore essential to accurate detection of GBS in maternal GBS surveillance, which is critical for optimizing neonatal GBS disease prevention and management.

**Supplementary Information:**

The online version contains supplementary material available at 10.1007/s10096-026-05483-8.

## Introduction

*Streptococcus agalactiae* (Group B *Streptococcus*, GBS) colonizes approximately 17% of pregnant women worldwide and remains a leading cause of neonatal sepsis and maternal infections despite routine screening and intrapartum antibiotic prophylaxis (IAP) [1–8]. Accurate detection of maternal colonization is therefore pivotal to prevent early-onset neonatal GBS disease. According to World Health Organization (WHO) global estimates, maternal GBS colonization affects approximately 17% of pregnant women worldwide, representing nearly 20 million pregnancies each year, contributing to more than 390,000 cases of invasive neonatal GBS disease, approximately 91,000 neonatal deaths, and over 50,000 stillbirths annually, with the disease burden disproportionately borne by low- and middle-income countries [9].

The CAMP test, which detects hemolysis mediated by the CAMP factor encoded by the *cfb* gene, has long been used for presumptive GBS identification. Additionally, the *cfb* gene is the most commonly used molecular target sequence for PCR-based GBS detection assays [10–13]. In recent years, a variety of nucleic acid amplification tests (NAATs) with distinct technical principles, including loop-mediated isothermal amplification (LAMP), real-time intrapartum PCR platforms, and other isothermal amplification technologies, have been increasingly adopted for point-of-care GBS detection [12, 14, 15]. These NAATs offer advantages such as shortened turnaround time and high analytical sensitivity, making them attractive alternatives to traditional culture-based screening. Notably, most of these molecular assays rely on the *cfb* gene as the single species-specific identification. Therefore, genetic alterations such as deletion or disruption of the *cfb* gene may compromise the detection sensitivity of not only conventional PCR assays but also isothermal NAATs, highlighting the necessity of multi-target molecular approaches for reliable GBS screening.

Although GBS detection assays have become increasingly standardized, emerging atypical lineages challenge their sensitivity. Rare CAMP-negative strains characterized by *cfb* deletions or disruptions can evade both the CAMP test and *cfb*-based PCR assays [16–19]. This diagnostic blind spot has important clinical implications, since undetected maternal GBS colonization may render IAP ineffective and significantly increase the risk of neonatal GBS infection. Enhancing the diagnostic sensitivity, particularly for atypical strains, is therefore critical for safeguarding maternal and neonatal health. Beyond addressing diagnostic escape, comprehensive molecular characterization of CAMP-negative GBS is essential to elucidate its epidemiological characteristics and pathogenic potential. Multilocus sequence typing (MLST) enables precise lineage assignment and facilitates comparison with globally recognized hypervirulent clones such as ST17 and ST19, which are strongly associated with invasive neonatal disease. Capsular serotyping is particularly relevant to the development of maternal GBS vaccines, as current vaccine candidates target specific capsular serotypes and require accurate epidemiological surveillance data to estimate vaccine coverage. In addition, profiling virulence-associated genes provides insight into the pathogenic repertoire of CAMP-negative GBS and helps clarify whether the absence of the *cfb* gene impairs, compensates, or coexists with other established virulence determinants. Comprehensive molecular analysis is necessary to determine whether CAMP-negative GBS represents merely a diagnostic variant or a distinct epidemiological sublineage with unique clinical implications.

Despite the increasing number of sporadic reports on *cfb*-deficient or CAMP-negative GBS, comprehensive molecular and clinical characterization of these strains remains limited, particularly in China, where large-scale epidemiological data are scarce. Most previous studies have focused primarily on the mechanisms of diagnostic escape, without systematically integrating analysis of antimicrobial resistance patterns, MLST, capsular serotyping, virulence gene profiles, and associated pregnancy outcomes in a defined screening population.

To address these knowledge gaps, three key questions were investigated in this study: (1) Do CAMP-negative GBS isolates represent sporadic diagnostic variants, or belong to specific clonal lineages with distinct molecular characteristics? (2) Does the absence of the *cfb* gene alter the virulence gene repertoire or antimicrobial resistance profile of GBS? (3)Are CAMP-negative GBS strains associated with distinct obstetric outcomes compared with conventional CAMP-positive isolates?

To answer these questions, we conducted a comprehensive analysis of CAMP-negative GBS isolates identified through a large maternal screening program (46,746 women) at a tertiary referral center in southern China. We characterized the molecular characteristics and clinical outcomes of CAMP-negative GBS isolates and compared them with CAMP-positive strains collected during the same period. The primary objectives of this study were to determine whether CAMP-negative GBS constitutes a distinct epidemiological lineages and to assess its implications for clinical GBS detection and perinatal management.

## Materials and methods

### Study setting and strain collection

From January 2017 to December 2021, all non-duplicate CAMP-negative GBS isolates identified at Guangzhou Medical University affiliated Guangzhou Women and Children’s Medical Center were enrolled in this study (*n* = 55), including 45 from pregnant women (for clinical outcome analysis) and 10 from non-pregnant outpatients (only for molecular and antimicrobial resistance characterization). For comparative purposes, 66 CAMP-positive GBS isolates collected during the same period were retrieved from the institutional strain collection. Duplicate isolates from the same patient and strains with incomplete microbiological data were excluded. This study was designed as a retrospective laboratory-based comparative analysis, and no individual matching was performed for the CAMP-positive control group.

### Strain identification

Vaginal and rectal swabs were inoculated into selective enrichment broth (Guangzhou Dijing Microbial Science and Technology, China) and incubated at 35 °C in 5% CO₂ for 18–24 h, followed by subculture on GBS chromogenic agar (Zhengzhou Antu Biological Engineering Co., Ltd., China) and 5% sheep blood agar. Suspected colonies were identified by the VITEK-2 Compact system (bioMérieux, France) and confirmed using MALDI-TOF MS (Bruker Microflex LT, Bremen, Germany). The CAMP test was performed on 5% sheep blood agar in the presence of *Staphylococcus aureus* ATCC 25,923 to produce β-hemolysin. *Streptococcus agalactiae* ATCC 13,813 and *Enterococcus faecalis* ATCC 29,212 served as positive and negative controls, respectively.

### Antimicrobial susceptibility testing

Antimicrobial susceptibility testing was performed according to the Clinical and Laboratory Standards Institute (CLSI) guidelines (M100, 30th edition, 2020) [20]. Disk diffusion was used for erythromycin, clindamycin, and tetracycline, whereas MIC values for penicillin, ampicillin, cefazolin, levofloxacin, vancomycin, and linezolid were determined using the E-test method. The antibiotics tested included penicillin, ampicillin, cefazolin, erythromycin, clindamycin, tetracycline, vancomycin, linezolid, and levofloxacin. *Staphylococcus aureus* ATCC 25,923 was used as a quality-control strain. The D-zone test was performed by placing erythromycin and clindamycin disks 12 mm apart (edge-to-edge) on 5% sheep blood Mueller–Hinton agar, incubated at 37 °C for 20–24 h in 5% CO₂. Results were interpreted according to CLSI breakpoints.

### Molecular characterization

#### Multilocus sequence typing (MLST)

MLST was performed by amplifying and sequencing 7 housekeeping genes (adhP, pheS, atr, glnA, sdhA, glcK, tkt) following standard protocols [19]. Sequence types (STs) were assigned via the PubMLST GBS database ( http://pubmlst.org/sagalactiae/ ).

#### Capsular serotyping

Capsular polysaccharide types were determined using multiplex PCR targeting *cpsL*, *cpsG*, *cpsN*, *cpsJ*, and *cpsI* genes [22]. PCR products were resolved on 1.5% high-resolution agarose-1000 gels (Life Technologies, USA) and visualized under UV illumination. Serotype results were confirmed using the Strep-B-Latex^®^ kit (SSI Diagnostica, Denmark).

#### Virulence gene detection

11 virulence-associated genes (*cfb*, *bac*, *fbsA*, *fbsB*, *lmb*, *cylE*, *hylB*, *pavA*, *scpB*, *neuC*, *pbp1A*) were detected by PCR. Reactions were performed in a SimpliAmp thermal cycler (Applied Biosystems, USA) under the following conditions: 95 °C for 5 min; 30 cycles of 95 °C for 30 s, 55 °C for 30 s, and 72 °C for 1 min; followed by a final extension at 72 °C for 5 min. Primer sequences were synthesized by BGI (Shenzhen, China), and their sequences are listed in Supplementary Table S1. PCR products were analyzed by electrophoresis on 1.5% agarose gels stained with ethidium bromide.

### Statistical analysis

Categorical data were expressed as counts and percentages. Inter-group differences were assessed using the χ² test or Fisher’s exact test when applicable. A P-value < 0.05 was considered statistically significant. Analyses were performed using SPSS version 26.0 (IBM, USA).

### Ethical approval

This study was approved by the Ethics Committee of Guangzhou Women and Children’s Medical Center (Approval No. 2025120A01). The study was conducted within the approved research period (2015–2024). Given the retrospective design and the use of anonymized clinical and microbiological data, the requirement for informed consent was waived by the ethics committee.

## Results

### Clinical findings

Of 46,746 pregnant women screened, 6,108 (13.1%) were GBS-positive, among which 45 (0.74%) isolates were identified as CAMP-negative. An additional 10 CAMP-negative isolates from non-pregnant outpatients were used solely for molecular typing and antimicrobial susceptibility testing, and were not included in pregnancy outcome analysis. Adverse pregnancy outcomes were documented in 19 of 45 colonized women (42.2%), including premature rupture of membranes (11/45, 24.4%), low birth weight (6/45, 13.3%), and preterm delivery (2/45, 4.4%). All 45 pregnant women colonized with CAMP-negative GBS were followed to delivery through hospital medical records, and no loss to follow-up occurred. For the CAMP-positive control, group, pregnancy outcome data were available for 60 women (6 CAMP-positive isolates were excluded because they were obtained from non-pregnant outpatients.), with preterm delivery in 2/60 (3.3%), PROM in 13/60 (21.7%), and low birth weight in 9/60 (15.0%). No statistically significant differences in the incidence of adverse pregnancy outcomes were observed between the CAMP-negative and CAMP-positive groups (Table 1).

**Table 1:** Comparison of pregnancy outcomes between women colonized with CAMP-negative and CAMP-positive GBS

Outcome	CAMP-negative GBS	CAMP-positive GBS	χ2 value	*P* value
	rate(%,*n* = 45)	rate(%,*n* = 60)		
PROM	24.44%(11/45)	21.67%(13/60)	0.17	0.68
Low birth weight	13.33%(6/45)	15.00%(9/60)	0.6	0.44
preterm delivery	4.44%(2/45)	3.33%(2/60)	0.0	1.0

Note: Data are presented as n (%). *PROM* premature rupture of membranes

### Microbiological and molecular characteristics

#### Antimicrobial susceptibility

**Fig. 1:**
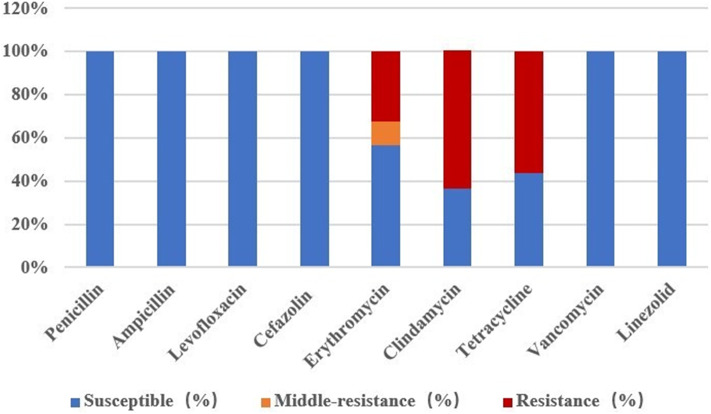
Antibiotic resistance profiles of CAMP-negative *Streptococcus agalactiae* strains. Resistance rates to erythromycin, clindamycin, and tetracycline were determined by disk diffusion according to CLSI guidelines

All 55 CAMP-negative GBS isolates were fully susceptible to penicillin, ampicillin, cefazolin, levofloxacin, vancomycin, and linezolid. Detailed antimicrobial susceptibility results are summarized in Table 2. Antimicrobial resistance was observed for erythromycin (18/55, 32.7%), tetracycline (31/55, 56.4%), and clindamycin (35/55, 63.6%). Some isolates showed clindamycin resistance without erythromycin resistance. D-zone testing showed that all erythromycin-resistant isolates were also resistant to clindamycin, and no inducible clindamycin resistance (iMLSB phenotype) was detected. The distribution of MLSB resistance phenotypes is summarized in Table S6. Among erythromycin-resistant isolates (*n* = 18), constitutive MLSB resistance was identified in 17 isolates, while no inducible clindamycin resistance was observed. A total of 19 CAMP-negative GBS isolates (34.5%) were classified as multidrug-resistant (MDR), defined as concurrent resistance to erythromycin, clindamycin, and tetracycline (Fig. 1).

**Table 2:** Antimicrobial susceptibility of 55 CAMP-negative Streptococcus agalactiae isolates

Antimicrobial agent	Susceptible *n* (%)	Intermediate *n* (%)	Resistant *n* (%)
Penicillin	55 (100)	0	0
Ampicillin	55 (100)	0	0
Levofloxacin	55 (100)	0	0
Cefazolin	55 (100)	0	0
Erythromycin	31 (56.4)	6(10.9)	18 (32.7)
Clindamycin	24 (43.6)	0	31 (56.4)
Tetracycline	20 (36.4)	0	35 (63.6)
Vancomycin	55 (100)	0	0
Linezolid	55 (100)	0	0

*AST* antimicrobial susceptibility testing

Susceptibility testing was performed using the disk diffusion method according to the Clinical and Laboratory Standards Institute (CLSI) guidelines (M100, 30th edition). Staphylococcus aureus ATCC 25923 was used as the quality control strain

### Sequence type distribution

**Fig. 2:**
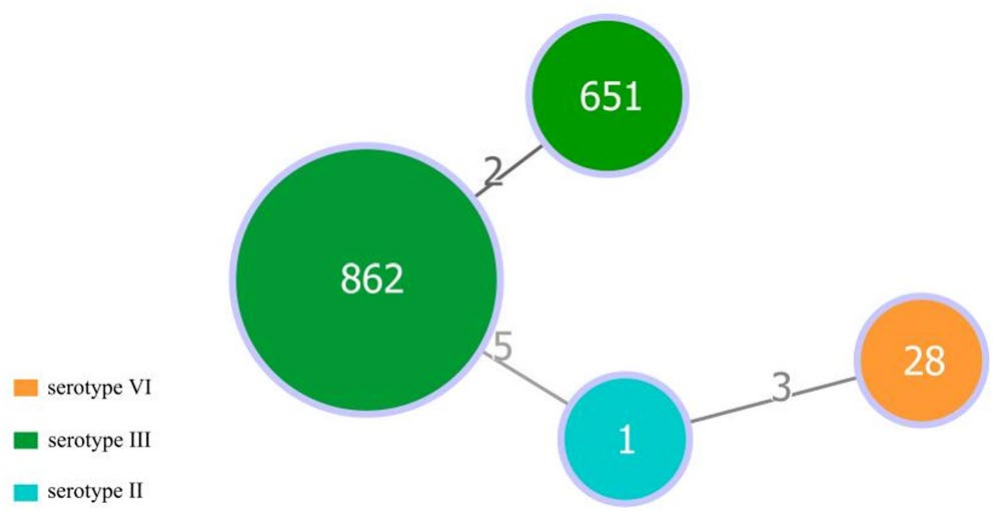
Minimum spanning tree analysis of sequence types (STs) and capsular serotypes among CAMP-negative GBS strains. *Not*e: Each circle represents a single ST, with the circle size proportional to the number of isolates of that ST. Different colors indicate distint capsular serotypes

MLST analysis identified four distinct sequence types (STs) among the 55 CAMP-negative GBS isolates: ST862 (50/55, 90.9%), ST651 (3/55, 5.5%), ST28 (1/55, 1.8%), and ST1 (1/55, 1.8%) (Fig. 2, Table S2). In contrast, 13 distinct STs were detected in the 66 CAMP-positive control isolates,, including ST19, ST23, ST17, ST10, ST862, ST529, ST12, ST1, ST890, and ST28 (Table S2, Figure S1). The proportion of ST862 isolates was significantly higher in the CAMP-negative group (90.9%) than in the CAMP-positive group (7.5%) (*p* < 0.05) (Table S2, Supplemental xls1).

### Capsular serotype distribution

Capsular serotyping revealed 53 of the 55 CAMP-negative GBS isolates (96.3%) belonged to serotype III, with one isolate each of serotype II and serotype VI. In the CAMP-positive control group, five predominant capsular serotypes were identified: III, Ia, V, Ib, and II (Table S3).

### Virulence gene profiles

**Fig. 3:**
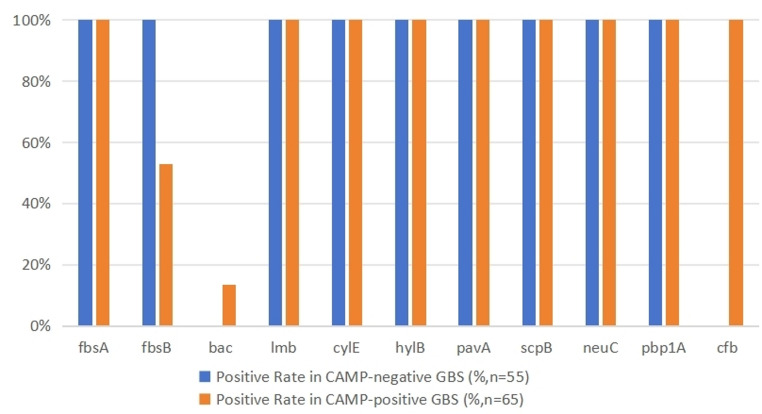
Distribution of 11 virulence-associated genes in CAMP-negative and CAMP-positive GBS strains

PCR detection of 11 virulence-associated genes showed that all CAMP-negative isolates lacked *cfb* and *bac* but carried *fbsA*, *fbsB*, *lmb*, *cylE*, *hylB*, *pavA*, *scpB*, *neuC* and *pbp1A* (100%). In contrast, all CAMP-positive isolates possessed *cfb*, while the positive rates of *fbsB* and *bac* were 53.0% and 13.6%, respectively (Fig. 3, Table S4).

## Discussion

This study provides the first comprehensive molecular and clinical characterization of CAMP-negative GBS isolates from pregnant and non-pregnant women in southern China. Our findings demonstrate that although CAMP-negative GBS is rare (accounting for only 0.74% of GBS-positive isolates in prenatal screening), these isolates demonstrated strong clonal relatedness, with the serotype III/ST862 being the absolute predominant genotype. This lineage-specific clustering may suggest a clonal expansion event, similar to the global dissemination of hypervirulent GBS lineages such as ST17 and ST19 [2, 8, 23]. The dominance of ST862 in our cohort contrasts with the global predominance of ST17 and ST19 which are strongly associated with invasive neonatal GBS disease, indicating potential regional heterogeneity in the population structure of GBS in Southern China. Further genomic sequencing studies are required to determine whether ST862 represents an emerging GBS sublineage or a regionally adapted clone with colonizing potential. Notably, all CAMP-negative isolates lacked the *cfb* gene encoding CAMP factor, but retained all other major virulence genes. This finding is consistent with the results of animal model studies, showing that CAMP factor is dispensable for mucosal colonization and systemic virulence [24, 25]. The retention of key adhesins and invasion-associated genes such as *fbsA*, *fbsB*, *scpB*, and *cylE* in CAMP-negative isolates suggests that loss of the *cfb* gene does not attenuate the pathogenic potential of GBS. These results support the hypothesis that CAMP-negative GBS strains maintain the ability to colonize the maternal genital tract and cause invasive disease in neonate despite the absence of CAMP factor activity.

The absence of *cfb* fully explains their negative CAMP reaction and highlights an important diagnostic blind spot. Reliance solely on the CAMP test or *cfb*-based PCR may cause false-negative identification of GBS [16–19]. These results reinforce prior observations that deletion or chromosomal rearrangement involving *cfb* can lead to diagnostic escape variants [18, 19]. Given that timely and accurate detection of maternal GBS colonization forms the cornerstone of intrapartum antibiotic prophylaxis (IAP), laboratories should adopt dual or multiplex molecular assays integrating alternative conserved targets such as *scpB* or *sip* [1, 12, 13, 26]. Integrated diagnostic workflows combining enrichment culture with multi-target PCR could improve the sensitivity and reliability of GBS detection, particularly for atypical strains.

In antimicrobial susceptibility testing, all CAMP-negative GBS isolates remained fully susceptible to β-lactams, vancomycin, and linezolid, which is consistent with global epidemiological data indicating that penicillin remains the first-line agent for IAP in GBS-positive pregnant women [27, 28]. However, relatively high resistance rates to macrolides (erythromycin, 32.7%) and lincosamides (clindamycin,56.4%) were observed in CAMP-negative GBS isolates, which has important clinical implications: macrolides and lincosamides are the most commonly used alternative antimicrobial agents for IAP in pregnant women with a reported penicillin allergy [14]. In the present study, no inducible clindamycin resistance was detected, as erythromycin-resistant isolates were also resistant to clindamycin, indicating constitutive MLSB resistance. This pattern suggests that macrolide–lincosamide resistance in these isolates is mainly mediated by constitutive MLSB resistance rather than inducible mechanisms. Verification of penicillin allergy status and continous regional surveillance resistance monitoring remain essential components of antimicrobial stewardship. Most MDR isolates belonged to the predominant serotype III/ST862 lineage, which is consistent with the strong clonal distribution observed among CAMP-negative isolates in this study, suggesting that antimicrobial resistance traits may be clonally transmitted in this lineage.

Clinically, 42.2% of pregnant women colonized with CAMP-negative GBS experienced adverse pregnancy outcomes, including premature rupture of membranes and preterm birth. Although no statistically significant differences were observed compared with CAMP-positive carriers, these outcomes are well recognized risk factors for neonatal GBS infection [2–6]. The potential for undetected CAMP-negative GBS colonization due to diagnostic limitations may compromise existing prevention strategies. Rapid intrapartum nucleic acid amplification tests (NAATs), such as LAMP or direct-from-swab PCR, have demonstrated excellent diagnostic performance and can serve as complementary screening tools for GBS detection in late pregnancy or during labor [15, 29], which may help identify undiagnosed CAMP-negative GBS carriers and optimize IAP implementation. The lack of statistical significance in adverse pregnancy outcomes between the two groups may be attributed to the small sample size of CAMP-negative isolates, which limited the statistical power of the analysis. Larger multicenter studies are needed to determine whether CAMP-negative GBS is associated with distinct obstetric or neonatal outcomes.

The 10 CAMP-negative isolates from non-pregnant women were included to strengthen the molecular and resistance profiling of this atypical GBS lineage, while all clinical and obstetric data were derived exclusively from the pregnant cohort, consistent with the study focus on maternal–neonatal diagnostic and management implications.

The lineage-specific clustering CAMP-negative GBS (serotype III/ST862) observed in this study highlights the need for continued genomic surveillance in southern China to monitor the spread of this lineage and determine whether it represents a stable regional clone or an emerging lineage with broader dissemination potential. From a public health perspective, these findings underscore a persistent gap between laboratory diagnostics and clinical prevention. Current guidelines from the US Centers for Disease Control and Prevention (CDC) and the American College of Obstetricians and Gynecologists (ACOG) [30, 31] recommend universal prenatal screening at 35–37 weeks of gestation but do not account for atypical *cfb*-deficient strains. European GBS screening policies remain heterogeneous, and the WHO has emphasized the urgent need for improved diagnostics and maternal vaccination to reduce the global GBS burden [9, 32]. Incorporating molecular surveillance for *cfb*-deficient strains into national screening frameworks could help refine prevention programs and ensure equitable protection. Future multicenter studies integrating genomic data and real-time molecular diagnostics could strengthen perinatal GBS prevention strategies across different epidemiological settings.

This study has several limitations. First, as a single-center study conducted in a tertiary referral hospital in Guangzhou, the findings may primarily reflect local epidemiology and should be interpreted cautiously when extrapolating to other regions. Second, whole-genome sequencing was not performed, which limits our ability to conduct detailed evolutionary analyses of *cfb* gene deletion events and to investigate the transmission dynamics of the serotype III/ST862 lineage. Third, CAMP-positive controls were not individually matched for demographic or clinical variables (e.g., age, gestational age, parity), which may have introduced residual confounding in the comparative analyses of pregnancy outcomes. Finally, the relatively small number of CAMP-negative isolates identified each year limited our ability to perform meaningful time-trend analysis of molecular characteristics and antimicrobial resistance profiles across the study years.

## Conclusion

In conclusion, our results reveal the hidden genetic diversity of GBS and highlight the diagnostic vulnerability posed by *cfb*-deficient CAMP-negative strains. These strains, although rare, exhibit a highly clonal distribution (serotype III/ST862) in southern China, retain full pathogenic potential, and can evade detection by conventional CAMP tests and single-target *cfb*-based PCR assays. Recognition of such atypical isolates is essential to maintaining the integrity of maternal GBS screening programs and to optimize neonatal GBS disease prevention. These findings have direct clinical implications for improving diagnostic microbiology and infection prevention in perinatal healthcare, emphasizing the need for integrated diagnostic approaches combining culture-based screening with multiplex PCR for accurate GBS detection.

## Supplementary Information

Below is the link to the electronic supplementary material.

Supplementary Material 1.

Supplementary Material 2.

Supplementary Material 3.

Supplementary Material 4.

Supplementary Material 5.

Supplementary Material 6.

Supplementary Material 7.

Supplementary Material 8.

Supplementary Material 9.

## Data Availability

All data supporting the findings of this study are available from the corresponding author upon reasonable request. No publicly archived datasets were generated during this study.
