# Supplementary material for: CAMP-negative group B Streptococcus in pregnant women: molecular and clinical features with implications for diagnostics and neonatal management
Source: Eur J Clin Microbiol Infect Dis. 2026 Mar 27;45(7):2025–32. doi: 10.1007/s10096-026-05483-8 (PMC13328311; doi:10.1007/s10096-026-05483-8)
Supplement: Supplementary file 1 — Supplementary Material 1. [file 10096_2026_5483_MOESM1_ESM.docx]

Supplementary Figures


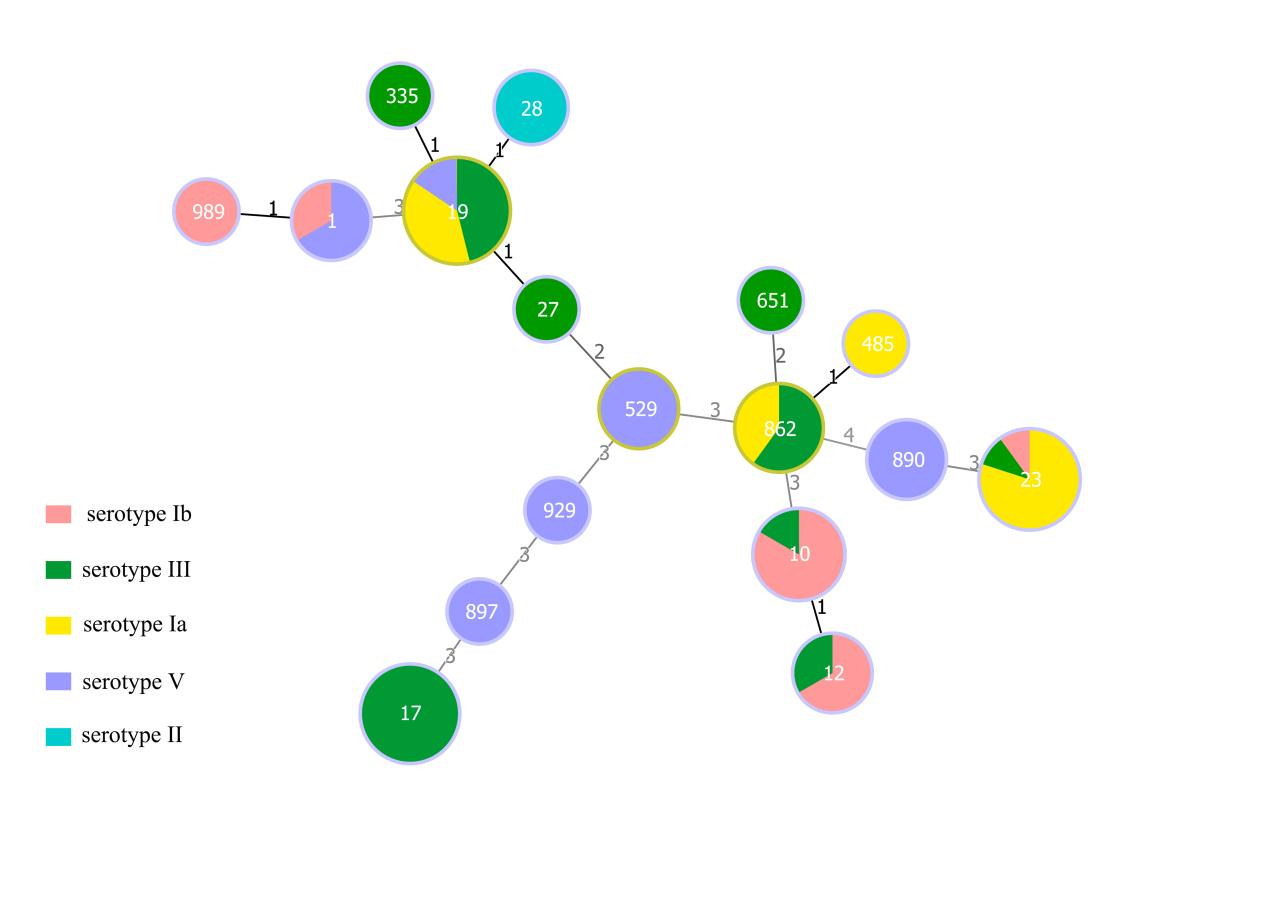


**Figure S1.** Distribution of STs and serotypes among CAMP-positive *Streptococcus agalactiae* strains by minimum spanning tree analysis
