# Supplementary material for: CAMP-negative group B Streptococcus in pregnant women: molecular and clinical features with implications for diagnostics and neonatal management
Source: Eur J Clin Microbiol Infect Dis. 2026 Mar 27;45(7):2025–32. doi: 10.1007/s10096-026-05483-8 (PMC13328311; doi:10.1007/s10096-026-05483-8)
Supplement: Supplementary file 2 — Supplementary Material 2. [file 10096_2026_5483_MOESM2_ESM.docx]

**Table S1** **Primers sequences of 11 virulence genes in two GBS reference genomes**

| **Category** | **Gene** | **Function/**  **annotation** | **NEM316(III)/A909(Ia)** | **Primer sequences (5'-3')**^a^ | **Product size(bp)** |
| --- | --- | --- | --- | --- | --- |
| **Adhesins** | *fbsA* | fibrinogen-binding protein FbsA | GBS1087 | F:CAACTTATAGGGAAAAATCCAC | 144 |
|  |  |  |  | R:AGTTAACATCGGTCTATTAGC |  |
|  | *pavA* | fibronectin-binding protein | GBS1263 | F:CCTATCGTACTATCCTTCCTGG | 408 |
|  |  |  |  | R:TCTCTAACTCACTTTGAACAC |  |
|  | *scpB* ***^b^*** | C5a peptidase | GBS1308 | F:TGCCATTGCGCTTATGTCTACGAGC | 574 |
|  |  |  |  | R:TCCTGACAAGATTCCTGACACGT |  |
|  | *fbsB* | fibrinogen-binding protein FbsB | GBS0850 | F:GCGATTGTGAATAGAATGAGTG | 149 |
|  |  |  |  | R:ACAGAAGCGGCGATTTCATT |  |
|  | *lmb* | laminin-binding protein | GBS1307 | F:CCCAAACAGCCTACGCAAG | 118 |
|  |  |  |  | R:TGCCTGCACCTGATTGGATC |  |
| **Invasins** | *hylB* | hyaluronate lyase | GBS1270 | F:TCCATTTAAAGCCCTTGGTG | 199 |
|  |  |  |  | R:GGCGCCAGTATAAGCAACAT |  |
|  | *cfb* | CAMP factor | GBS2000 | F:TAGGGGAAAAGAAAGCGCTTGA | 614 |
|  |  |  |  | R:GCCTTTACATCGTTAACTTGA |  |
|  | *cylE* | Cytolysin E, cyl^c^ E | GBS0651 | F:TGACATTTACAAGTGACGAAG | 248 |
|  |  |  |  | R:TTGCCAGGAGGAGAATAGGA |  |
| **Immune evasins** | *bac* | C-β protein | SAK0186 | F:CTATTTTTGATATTGACAATGCAA | 592 |
|  |  |  |  | R:GTCGTTACTTCCTTGAGATGTAAC |  |
|  | *neuC* | neu gene cluster C | GBS1235 | F:AGGGGATCGTTACGAAATGT | 463 |
|  |  |  |  | R:AGCCTTTGCAATATCATCAGA |  |
|  | *pbp1A* | penicillin-binding protein 1A | GBS0288 | F:CAGACCGCGATTGGGGTTCTA | 552 |
|  |  |  |  | R:CTGCTTTAGTACCAGTACCA |  |

a. F, forward; R, reverse.

b. dual roles of both an adhesin and an immune system evading gene.

c. although primarily an invasin, cyl is capable of damaging phagocytes and hence also have a role in immune system evasion

**Table 2** **MLST analysis of CAMP-negative GBS strains**

| **MLST** | **CAMP-negative GBS（n=55）** |  | **CAMP-positive GBS(n=66)** | **χ^2^ value** | ***P* value** |
| --- | --- | --- | --- | --- | --- |
|  | **Rate %（n=55）** |  | **Rate %（n=66）** |  |  |
| ST1 | 1.8%(1/55) |  | 4.55% (3/66)  9.09% (6/66)  4.55% (3/66)  13.64%(9/66)  19.70%(13/66)  15.15%(10/66)  1.52%(1/66)  3.03(2/66)  1.52%(1/66)  1.52%(1/66)  4.55% (3/66)  1.52%(1/66)  7.58(5/66)  1.52%(1/66)  4.55%(3/66)  1.52%(1/65)  1.52%(1/66)  1.52%(1/66) | 0.116 | 0.734 |
| ST10 | 0 |  |  | 3.577 | 0.059 |
| ST12 | 0 |  |  | 1.054 | 0.305 |
| ST17 | 0 |  |  | 6.358 | 0.016 |
| ST19 | 0 |  |  | 12.336 | ＜0.001 |
| ST23 | 0 |  |  | 7.327 | 0.007 |
| ST27 | 0 |  |  | - | 1.000 |
| ST28 | 1.8%(1/55) |  |  | ＜0.001 | 1.000 |
| ST335 | 0 |  |  | - | 1.000 |
| ST485 | 0 |  |  | - | 1.000 |
| ST529 | 0 |  |  | 1.054 | 0.305 |
| ST651 | 5.5%(3/55) |  |  | 0.463 | 0.496 |
| ST862 | 90.9%(50/55) |  |  | 83.100 | ＜0.001 |
| ST882 | 0 |  |  | - | 1.000 |
| ST890 | 0 |  |  | 1.054 | 0.305 |
| ST897 | 0 |  |  | - | 1.000 |
| ST929 | 0 |  |  | - | 1.000 |
| ST989 | 0 |  |  | - | 1.000 |
| NT | / |  | 1.52%(1/66) |  |  |

Note: A dash(-) denotes Fisher's exact probability test; χ2 values are not applicable.

Chi-square (χ²) or Fisher's exact test was used for group comparison.

**Table 3 Serotyping analysis of CAMP-negative GBS strains**

| **Serotype** | **CAMP-negative GBS（n=55）** |  | **CAMP-positive GBS（n=66）** | **χ^2^值** | ***P*值** |
| --- | --- | --- | --- | --- | --- |
|  | **Rate (%, n=55)** |  | **rate（%, n=66）** |  |  |
| Ia | 0 |  | 25.76%(17/66) | 19.338 | ＜0.001 |
| Ib | 0 |  | 15.15%(10/66) | 8.828 | 0.003 |
| II | 1.82%(1/55) |  | 3.03%(2/66) | 0.046 | 0.830 |
| III | 96.36%(53/55) |  | 37.88%(25/66) | 36.864 | ＜0.001 |
| V | 0 |  | 18.18%(12/66) | - | 0.190 |
| VI | 1.82%(1/55) |  | 0 | - | 1.000 |

Note: - indicates Fisher's exact probability test, χ2 value is not applicable.

**Table S4 Comparison of virulence gene distribution between CAMP-negative and CAMP-positive Streptococcus agalactiae strains**

| **Gene** | **CAMP-negative GBS** |  | **CAMP-positive GBS** | **χ2 value** | ***P* value** |
| --- | --- | --- | --- | --- | --- |
|  | **rate（%,n=55）** |  | **rate（%,n=66）** |  |  |
| fbsA | 100%(55/55) |  | 100.00%(66/66) | - | -b |
| fbsB | 100%(55/55) |  | 53.03%(35/66) | 58.785 | ＜0.001 |
| bac | 0.00%(0/55) |  | 13.64%(9/66) | 6.358 | 0.015 |
| lmb | 100%(55/55) |  | 10000%(66/66) | - | -b |
| cylE | 100%(55/55) |  | 100.00%(66/66) | - | -b |
| hylB | 100%(55/55) |  | 100.00%(66/66) | - | -b |
| pavA | 100%(55/55) |  | 100.00%(66/66) | - | -b |
| scpB | 100%(55/55) |  | 100.00%(66/66) | - | -b |
| nevC | 100%(55/55) |  | 100.00%(66/66) | - | -b |
| pbp1A | 100%(55/55) |  | 100.00%(66/66) | - | -b |
| cfb | 0.00%(0/55) |  | 100.00%(66/66) | 120.000 | ＜0.001 |

Note: -b means that the p-value cannot be calculated because there is no corresponding result.

**Table S5 Correlation between serotypes and MLST types among CAMP-negative and CAMP-positive Streptococcus agalactiae strains**

| **Serotype** | **STs in CAMP-negative GBS (%, n=55)** | **STs in CAMP-positive GBS**  **(%, n=66)** |
| --- | --- | --- |
| Ia | 0 | ST23 (12.12%, 8/66), ST19 (7.58%,5/66), ST862 (3.03%,2/66), ST882 (1.52%,1/66), ST485 (1.52%,1/66) |
| Ib | 0 | ST10(7.58%,5/66), ST12 (3.03%,2/66), ST23 (1.52%,1/66), ST1(1.52%,1/66), ST989 (1.52%,1/66) |
| II | ST1(2.22%,1/55) | ST28 (3.03%,2/66) |
| III | ST862 (90.90%,50/55), ST651 (5.45%,3/55) | ST17(13.64%,9/66), ST19(9.09%,6/66), ST862(4.55%,3/66), ST12(1.52%,1/66), ST23 (1.52%,1/66), ST27(1.52%,1/66), ST651 (1.52%,1/66), ST335 (1.52%,1/66), ST10 (1.52%,1/66), NT(1.52%,1/66) |
| V | 0 | ST1(3.03%,2/66), ST19(3.03%,2/66), ST529 (1.52%,3/66), ST890 (4.55%,3/66), ST897(1.52%,1/66), ST929(1.52%,1/66), |
| VI | ST28 (2.22%,1/55) | 0 |
