# Supplementary material for: CAMP-negative group B Streptococcus in pregnant women: molecular and clinical features with implications for diagnostics and neonatal management
Source: Eur J Clin Microbiol Infect Dis. 2026 Mar 27;45(7):2025–32. doi: 10.1007/s10096-026-05483-8 (PMC13328311; doi:10.1007/s10096-026-05483-8)
Supplement: Supplementary file 3 — Supplementary Material 3. [file 10096_2026_5483_MOESM3_ESM.docx]

**Table S2** **MLST analysis of CAMP-negative GBS strains**

| **MLST** | **CAMP-negative GBS（n=55）** |  | **CAMP-positive GBS(n=66)** | **χ^2^ value** | ***P* value** |
| --- | --- | --- | --- | --- | --- |
|  | **Rate %（n=55）** |  | **Rate %（n=66）** |  |  |
| ST1 | 1.8%(1/55) |  | 4.55% (3/66)  9.09% (6/66)  4.55% (3/66)  13.64%(9/66)  19.70%(13/66)  15.15%(10/66)  1.52%(1/66)  3.03(2/66)  1.52%(1/66)  1.52%(1/66)  4.55% (3/66)  1.52%(1/66)  7.58(5/66)  1.52%(1/66)  4.55%(3/66)  1.52%(1/65)  1.52%(1/66)  1.52%(1/66) | 0.116 | 0.734 |
| ST10 | 0 |  |  | 3.577 | 0.059 |
| ST12 | 0 |  |  | 1.054 | 0.305 |
| ST17 | 0 |  |  | 6.358 | 0.016 |
| ST19 | 0 |  |  | 12.336 | ＜0.001 |
| ST23 | 0 |  |  | 7.327 | 0.007 |
| ST27 | 0 |  |  | - | 1.000 |
| ST28 | 1.8%(1/55) |  |  | ＜0.001 | 1.000 |
| ST335 | 0 |  |  | - | 1.000 |
| ST485 | 0 |  |  | - | 1.000 |
| ST529 | 0 |  |  | 1.054 | 0.305 |
| ST651 | 5.5%(3/55) |  |  | 0.463 | 0.496 |
| ST862 | 90.9%(50/55) |  |  | 83.100 | ＜0.001 |
| ST882 | 0 |  |  | - | 1.000 |
| ST890 | 0 |  |  | 1.054 | 0.305 |
| ST897 | 0 |  |  | - | 1.000 |
| ST929 | 0 |  |  | - | 1.000 |
| ST989 | 0 |  |  | - | 1.000 |
| NT | / |  | 1.52%(1/66) |  |  |

Note: A dash(-) denotes Fisher's exact probability test; χ2 values are not applicable.
