# Supplementary material for: CAMP-negative group B Streptococcus in pregnant women: molecular and clinical features with implications for diagnostics and neonatal management
Source: Eur J Clin Microbiol Infect Dis. 2026 Mar 27;45(7):2025–32. doi: 10.1007/s10096-026-05483-8 (PMC13328311; doi:10.1007/s10096-026-05483-8)
Supplement: Supplementary file 4 — Supplementary Material 4. [file 10096_2026_5483_MOESM4_ESM.docx]

**Table 3 Serotyping analysis of CAMP-negative GBS strains**

| **Serotype** | **CAMP-negative GBS（n=55）** |  | **CAMP-positive GBS（n=66）** | **χ^2^值** | ***P*值** |
| --- | --- | --- | --- | --- | --- |
|  | **Rate (%, n=55)** |  | **rate（%, n=66）** |  |  |
| Ia | 0 |  | 25.76%(17/66) | 19.338 | ＜0.001 |
| Ib | 0 |  | 15.15%(10/66) | 8.828 | 0.003 |
| II | 1.82%(1/55) |  | 3.03%(2/66) | 0.046 | 0.830 |
| III | 96.36%(53/55) |  | 37.88%(25/66) | 36.864 | ＜0.001 |
| V | 0 |  | 18.18%(12/66) | - | 0.190 |
| VI | 1.82%(1/55) |  | 0 | - | 1.000 |

Note: - indicates Fisher's exact probability test, χ2 value is not applicable.
