# Supplementary material for: CAMP-negative group B Streptococcus in pregnant women: molecular and clinical features with implications for diagnostics and neonatal management
Source: Eur J Clin Microbiol Infect Dis. 2026 Mar 27;45(7):2025–32. doi: 10.1007/s10096-026-05483-8 (PMC13328311; doi:10.1007/s10096-026-05483-8)
Supplement: Supplementary file 5 — Supplementary Material 5. [file 10096_2026_5483_MOESM5_ESM.docx]

**Table S5 Correlation between serotypes and MLST types among CAMP-negative and CAMP-positive Streptococcus agalactiae strains**

| **Serotype** | **STs in CAMP-negative GBS (%, n=55)** | **STs in CAMP-positive GBS**  **(%, n=66)** |
| --- | --- | --- |
| Ia | 0 | ST23 (12.12%, 8/66), ST19 (7.58%,5/66), ST862 (3.03%,2/66), ST882 (1.52%,1/66), ST485 (1.52%,1/66) |
| Ib | 0 | ST10(7.58%,5/66), ST12 (3.03%,2/66), ST23 (1.52%,1/66), ST1(1.52%,1/66), ST989 (1.52%,1/66) |
| II | ST1(2.22%,1/55) | ST28 (3.03%,2/66) |
| III | ST862 (90.90%,50/55), ST651 (5.45%,3/55) | ST17(13.64%,9/66), ST19(9.09%,6/66), ST862(4.55%,3/66), ST12(1.52%,1/66), ST23 (1.52%,1/66), ST27(1.52%,1/66), ST651 (1.52%,1/66), ST335 (1.52%,1/66), ST10 (1.52%,1/66), NT(1.52%,1/66) |
| V | 0 | ST1(3.03%,2/66), ST19(3.03%,2/66), ST529 (1.52%,3/66), ST890 (4.55%,3/66), ST897(1.52%,1/66), ST929(1.52%,1/66), |
| VI | ST28 (2.22%,1/55) | 0 |
