# Supplementary material for: CAMP-negative group B Streptococcus in pregnant women: molecular and clinical features with implications for diagnostics and neonatal management
Source: Eur J Clin Microbiol Infect Dis. 2026 Mar 27;45(7):2025–32. doi: 10.1007/s10096-026-05483-8 (PMC13328311; doi:10.1007/s10096-026-05483-8)
Supplement: Supplementary file 7 — Supplementary Material 7. [file 10096_2026_5483_MOESM7_ESM.docx]

**Table S4 Comparison of virulence gene distribution between CAMP-negative and CAMP-positive Streptococcus agalactiae strains**

| **Gene** | **CAMP-negative GBS** |  | **CAMP-positive GBS** | **χ2 value** | ***P* value** |
| --- | --- | --- | --- | --- | --- |
|  | **rate（%,n=55）** |  | **rate（%,n=66）** |  |  |
| fbsA | 100%(55/55) |  | 100.00%(66/66) | - | -b |
| fbsB | 100%(55/55) |  | 53.03%(35/66) | 58.785 | ＜0.001 |
| bac | 0.00%(0/55) |  | 13.64%(9/66) | 6.358 | 0.015 |
| lmb | 100%(55/55) |  | 100.00%(66/66) | - | -b |
| cylE | 100%(55/55) |  | 100.00%(66/66) | - | -b |
| hylB | 100%(55/55) |  | 100.00%(66/66) | - | -b |
| pavA | 100%(55/55) |  | 100.00%(66/66) | - | -b |
| scpB | 100%(55/55) |  | 100.00%(66/66) | - | -b |
| nevC | 100%(55/55) |  | 100.00%(66/66) | - | -b |
| pbp1A | 100%(55/55) |  | 100.00%(66/66) | - | -b |
| cfb | 0.00%(0/55) |  | 100.00%(66/66) | 120.000 | ＜0.001 |

Note: -b means that the p-value cannot be calculated because there is no corresponding result.
