# Supplementary material for: CAMP-negative group B Streptococcus in pregnant women: molecular and clinical features with implications for diagnostics and neonatal management
Source: Eur J Clin Microbiol Infect Dis. 2026 Mar 27;45(7):2025–32. doi: 10.1007/s10096-026-05483-8 (PMC13328311; doi:10.1007/s10096-026-05483-8)
Supplement: Supplementary file 8 — Supplementary Material 8. [file 10096_2026_5483_MOESM8_ESM.docx]

**Table S6 Distribution of MLSB resistance phenotypes**

| **Phenotype** | **Number of isolates** | **Percentage** |
| --- | --- | --- |
| cMLSB | 17 | 30.9% |
| iMLSB | 0 | 0 |
| M phenotype | 0 | 0 |

Note: D-zone testing was performed using erythromycin and clindamycin disks according to CLSI M100 (30th edition, 2020).
